# Integrated HIV and STIs response: Trends in syphilis incidence and uptake of oral pre-exposure prophylaxis in Zambia

**DOI:** 10.4102/jphia.v16i1.1306

**Published:** 2025-07-10

**Authors:** Kutha Banda, Nicholus C. Sande, Chipwaila C. Chunga, Belia Longwe, Kayawe Nkumbwa, Madaliso Silondwa, Nsanzya Maambo, Japhet Michelo, Prudence Haimbe, Trevor Mwamba, Hilda Shakwelele, Sandra Chilengi-Sakala, Ireen Bwalya

**Affiliations:** 1 Clinton Health Access Initiative, Lusaka, Zambia; 2 Women in Global Health Zambia, Lusaka, Zambia; 3 National AIDS Council, Lusaka, Zambia; 4 Department of Research, National Health Research Authority, Lusaka, Zambia; 5 Department of Policy and Planning, National AIDS Council, Lusaka, Zambia; 6 National Health Research Authority, Lusaka, Zambia; 7 Department of Public Health, Clinton Health Access Initiative, Lusaka, Zambia; 8 Ministry of Health, Lusaka, Zambia

**Keywords:** syphilis, pre-exposure prophylaxis, incidence, sexual health, trend analysis

## Abstract

**Background:**

Pre-exposure prophylaxis (PrEP) for human immunodeficiency virus (HIV) was introduced in Zambia to prevent transmission, but it does not protect against sexually transmitted infections (STIs) such as syphilis. Globally, STIs have risen alongside PrEP rollout, posing significant public health concerns that require urgent attention and targeted intervention strategies.

**Aim:**

We examined trends in syphilis incidence and assessed its association with PrEP use, given the increasing global burden of STIs, including among PrEP users between 2021 and 2023.

**Setting:**

Among individuals in Zambia.

**Methods:**

A retrospective database analysis of secondary data was conducted using District Health Information Software 2 (DHIS2), the Ministry of Health’s primary data system. Microsoft^®^ Excel and Stata were used for descriptive statistics and regression analysis to examine potential associations.

**Results:**

From 2021 to 2023, syphilis cases (199 273) and PrEP initiation (436 460) increased annually. Syphilis cases rose from 22% to 46%, while PrEP initiation grew from 22% to 48%. We found a positive association between syphilis incidence and PrEP initiation, with each unit increase in PrEP initiation corresponding to a 0.33-unit rise in syphilis cases (*p* < 0.001; 95% confidence interval [CI]: 0.25–0.4), underscoring this relationship.

**Conclusion:**

This analysis found a significant positive link between syphilis incidence and PrEP initiation, emphasising the need for integrated HIV and STI management to enhance public health interventions.

**Contribution:**

This study provides valuable insights for policy and programme implications; it highlights the importance of integrating STI prevention into HIV prevention service delivery; an integrated approach is critical to ensure that the country does not regress the achievements made towards HIV epidemic control.

## Introduction

Sexually transmitted infections (STIs) remain a persistent public health concern worldwide, posing significant challenges to global healthcare systems. Despite being easily diagnosed and treated, syphilis remains widespread, with six million new cases annually among individuals aged 15 years to 49 years, incipiently in middle-income countries.^1^

Although several advances in prevention and treatment strategies have been implemented, the incidence of STIs continues to rise. It is estimated that 374 million new STIs are acquired annually globally.^2^ These include curable sexually transmitted diseases (STDs) such as chlamydia, gonorrhoea, syphilis and Trichomonas vaginalis.^2^

In 2021, the worldwide prevalence of syphilis was estimated at approximately 70.5 million cases, with a 95% uncertainty interval ranging from 54.9 million to 88.2 million cases. Central sub-Saharan Africa experienced the highest burden, reporting 4623 cases. Over the period from 1990 to 2021, syphilis incidence showed a notable increase; increasing from 11 974 753.64 (95% uncertainty interval (UI): 8 932 531.75–1 555 536.31) in 1990 to 18 696 009.14 (95% UI: 114 033 725.79–24 331 643.87) in 2021, demonstrating an increase of 6 721 255.50 (95% UI: 5 101 194.04–8 776 274.56). This highlights the ongoing public health challenge posed by this infection. Despite advancements in diagnosis and treatment, syphilis remains a major concern, particularly in regions with limited healthcare resources.^3^

The Lancet Child and Adolescent Health report further added that since 2016, rates of gonorrhoea have increased by 45%, syphilis by 52% and congenital syphilis by 235%. Young people aged 15–24 years accounted for 53% of new STIs in 2020; 62% of new chlamydia cases were in adolescents, and the greatest rises in both syphilis and gonorrhoea were in women aged 15–24 years.^2^

Low- and middle-income countries (LMICs) generally have higher burdens of syphilis, with the African region consistently bearing the highest burden.^4,5^ In low-income countries, the combined prevalence of human immunodeficiency virus (HIV), hepatitis B virus (HBV), hepatitis C virus (HCV) and syphilis surpassed the global averages, with estimates of 5.2% (1.6% – 10.5%) for HIV, 6.6% (5.4% – 7.9%) for HBV, 2.7% (1.6% – 4.1%) for HCV and 3.3% (2.2% – 4.6%) for syphilis.^6^ Similarly, in lower-middle-income countries, the pooled prevalence rates for these infections were also higher than the worldwide levels, reported as 2.9% (0.8% – 6.1%) for HIV, 4.9% (3.8% – 6.1%) for HBV, 2.3% (1.2% – 3.6%) for HCV and 1.5% (1.0% – 2.2%) for syphilis.^7^

Syphilis is one of the most prevalent STIs in Zambia with the 2016 Zambia Population-based HIV Impact Assessment (ZAMPHIA) national household survey giving a prevalence of syphilis of 3.5% among adults aged 15–49 years with the prevalence of HIV/active syphilis co-infection of 1.5%.^8^ Furthermore, the 2018 Demographic Health Survey report found that 5% of women and 8% of men reported to have an STI,^9^ while Solomon et al.^10^ found an even higher co-infection rate of HIV and syphilis as high as 40.5%.

### Syphilis and human immunodeficiency virus

Recent evidence has demonstrated a strong association between STIs, such as syphilis, and HIV infection across various settings. A retrospective cohort study examining STI incidence among individuals using pre-exposure prophylaxis (PrEP) found a significant increase in STI rates in the 12 months following PrEP initiation.^11^ These findings highlight the need for comprehensive STI screening and prevention strategies alongside PrEP use to mitigate the risk of co-infections.

The introduction of PrEP, which is a biomedical prevention method, has demonstrated remarkable efficacy in reducing the transmission of HIV among at-risk populations (men who have sex with men [MSM], people who inject drugs and female sex workers). Zambia implemented PrEP in 2016, with a gradual increase in uptake during the initial years. By September 2018, 3626 individuals had initiated PrEP; however, this number increased more than sixfold to 23 327 by September 2019, with services available at 728 sites across all 10 provinces. Over the first 3 years of the programme, a total of 26 953 individuals initiated PrEP, of whom 31% were from key and priority populations.^12^

Even though PrEP can offer efficient protection against HIV, it is not protective against blood-borne infections or other STIs.^11^ Sexually transmitted infection rates are rising globally, especially among PrEP users in high-income countries, and a similar trend is observed in LMICs with the limited data available.^13^

Studies have found that PrEP users tend to have higher rates of STIs, partly because of behavioural factors such as condomless sex.^14,15,16^ In particular, syphilis has been identified as a significant STI among PrEP users, with incidence rates increasing over time. Research indicates that within 6 months of PrEP initiation, approximately 3.3% of STI cases were attributed to syphilis, and this proportion rose to 5.5% after 12 months.^17^ These findings underscore the importance of integrating routine STI screening, risk-reduction counselling and behavioural interventions into PrEP programmes to mitigate the risk of co-infections.

If left untreated, syphilis can result in life-threatening consequences and increases the possibility of contracting HIV.^18^ Since 2015, the World Health Organization (WHO) has recommended PrEP for high HIV-risk individuals. While initially widespread in high-income countries, PrEP utilisation is expanding to LMIC nations, with over 60 countries, including 20 in Africa, adopting national policies.^19^ Following this evidence of PrEP efficacy in lowering HIV acquisition in high-risk populations, PrEP was launched in Zambia in 2016.^12^ Emerging evidence suggests a link between STIs, such as syphilis, and PrEP initiation. However, limited research has examined this relationship in Zambia. As the country scales up PrEP services in its efforts towards HIV epidemic control, understanding its association with syphilis incidence is crucial for optimising programme implementation and informing targeted public health interventions.

### Aim

With the increase in observed syphilis cases, this article demonstrates the trends in syphilis incidence and PrEP initiation, and further investigates the associations between PrEP utilisation and syphilis incidence in Zambia from 2021 to 2023.

## Research methods and design

### Study design

This was a secondary data analysis using retrospective analysis of programme data on syphilis incidence and PrEP uptake from 2021 to 2023. The secondary data utilised is from the Ministry of Health’s Health Management Information System (HMIS) for the period 2021–2023 for all the 10 provinces in Zambia.

### Setting

We extracted the raw data from the DHIS2 system ourselves and did not use any already extracted data. The data were collected from health facilities providing HIV prevention services, including PrEP, and STI diagnosis and treatment. These facilities range from primary healthcare centres to tertiary hospitals, covering both rural and urban settings.

Zambia’s healthcare system operates within diverse geographical and socio-economic landscapes. The 10 provinces encompass a mix of densely populated urban areas with high STI prevalence and rural regions where access to healthcare services is often limited. Urban centres include informal settlements characterised by high population density. Conversely, peri-urban and rural areas have lower population densities but face unique healthcare access challenges.

### Study population

The analysis included data for the general population from facilities that provide PrEP and STI services across all 10 provinces of Zambia, encompassing all age groups and sexes.

### Data collection

This analysis was based on secondary data obtained from District Health Information Software 2 (DHIS2); the primary data management system used by the Ministry of Health, which aggregates health service data at the facility level. Data extraction was conducted by officials from the Monitoring and Evaluation Unit, covering the period from 2021 to 2023. The dataset included records from various levels of healthcare facilities, ranging from primary to tertiary institutions.

Specifically, all data on STI incidence and PrEP uptake among the general population were extracted onto an Microsoft^®^ Excel sheet.

### Data analysis

The data was extracted in Microsoft^®^ Excel, where descriptive analysis was performed. We further exported the data to Stata and used linear regression analysis to investigate the association between syphilis incidence and PrEP initiation.

### Ethical considerations

A waiver approval was granted by the National Health Research Authority (No. NHRA-003/11/11/2024) as data extracted were aggregated at facility level with no identifiers. In addition, permission was obtained from the Ministry of Health to access DHIS2 data. Confidentiality and anonymity were ensured, as the data lacked client identifiers and were presented in aggregate form.

## Results

**FIGURE 1:**
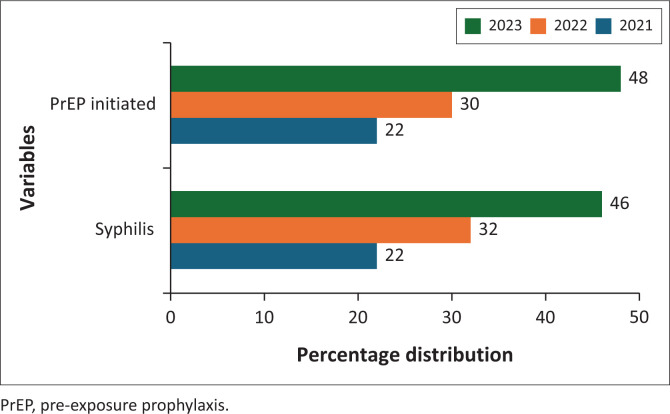
Trends in Syphilis incidence and pre-exposure prophylaxis initiation in Zambia (2021–2023).

Figure 1 presents the percentage distribution of syphilis incidence and PrEP initiation in Zambia from 2021 to 2023. During this period, syphilis cases increased annually, with 22% of cases recorded in 2021, 32% in 2022 and 46% in 2023. A similar trend was observed in PrEP initiation, with 22% of individuals initiating PrEP in 2021, rising to 30% in 2022 and 48% in 2023. These findings highlight a concurrent increase in both syphilis incidence and PrEP uptake over the study period.

Syphilis incidence was recorded across all provinces in Zambia from 2021 to 2023 (Table 1). Notably, provinces with higher PrEP initiation rates also experienced a significant rise in syphilis cases. For example, Lusaka province had the highest syphilis incidence in 2021 (7270 cases), while PrEP initiation stood at 10 134, ranking third among provinces. Over the following years, syphilis cases in Lusaka increased to 9235 in 2022 and 12 111 in 2023, with a concurrent rise in PrEP initiation, which more than tripled to 23 602 in 2022 and 33 202 in 2023.

**TABLE 1:** Incidence of syphilis and pre-exposure prophylaxis initiation between 2021 and 2023 by province.

Province	Syphilis incidence			PrEP initiated		
	2021	2022	2023	2021	2022	2023
Central	6611	9322	13 517	8496	10 806	35 507
Copperbelt	4916	6584	10 366	19 110	18 691	49 043
Eastern	3954	4893	8471	8395	9281	15 443
Luapula	5016	7117	9535	3674	6177	5156
Lusaka	7270	9235	12 111	10 134	23 602	33 202
Muchinga	2038	2733	3691	5462	5929	5201
Northern	2904	4553	5820	8331	8625	13 800
Northwestern	4074	6979	9876	2934	4642	5257
Southern	4317	7271	11 770	21 168	32 299	33 370
Western	3692	4505	6426	7189	11 570	14 216

**Total**	**44 792**	**63 192**	**91 583**	**94 893**	**131 622**	**210 195**

PrEP, pre-exposure prophylaxis.

In contrast, Muchinga province had the lowest syphilis incidence in 2021 (2038 cases), with PrEP initiation at 5462. Although syphilis cases in Muchinga increased over time, the growth was relatively lower than in other provinces, reaching 2733 in 2022 and 3691 in 2023. Similarly, PrEP initiation showed a modest increase to 5929 in 2022 before slightly declining to 5201 in 2023. These trends suggest a potential association between PrEP initiation and syphilis incidence, warranting further investigation into contributing factors.

We further disaggregated the data by rural and urban; overall, rural facilities had the highest proportion of syphilis cases across the 3 years, accounting for 63% of all the syphilis cases as compared to 37% in urban facilities (Table 2). The table also shows 44 792 reported cases of syphilis in 2021, with 61% originating from rural facilities and 39% from urban facilities. During the same year, PrEP initiation totalled 94 893, with 54% from urban facilities and 46% from rural facilities. The following year, syphilis cases rose to 63 169, with 63% from rural facilities and reduced to 37% in urban facilities, while PrEP initiation increased to 131 622, with 51% from urban and 49% from rural facilities. By 2023, syphilis cases had further increased to 91 310, with 65% from rural facilities and reduced to 35% in urban facilities. Pre-exposure prophylaxis initiation also saw a rise to 210 195, with 53% from urban facilities and 47% from rural facilities.

**TABLE 2:** Incidence of syphilis and pre-exposure prophylaxis initiation from 2021 to 2023 in Zambia by rural and urban.

Year	Facility type	Syphilis		PrEP initiated	
		*n*	%	*n*	%
2021	Rural	27 408	61	43 350	46
	Urban	17 384	39	51 543	54
	Total	44 792	100	94 893	100
2022	Rural	39 599	63	64 023	49
	Urban	23 593	37	67 599	51
	Total	63 169	100	131 622	100
2023	Rural	59 212	65	99 450	47
	Urban	32 371	35	110 745	53
	Total	91 310	100	210 195	100

PrEP, pre-exposure prophylaxis.

Table 2 also shows that overall, PrEP initiation increased across the years in both rural and urban areas, with urban facilities having a slightly higher percentage (53%) of clients initiated as compared to 47% in rural areas. When disaggregated by year, the Table shows that 54% of clients initiated on PrEP were from urban areas compared to 46% from rural areas. This slightly dropped to 51% for urban areas compared to 49% for rural areas in the following year. In 2023, the percentage of PrEP enrolment in urban areas increased to 53% compared to 47% in rural areas.

**FIGURE 2:**
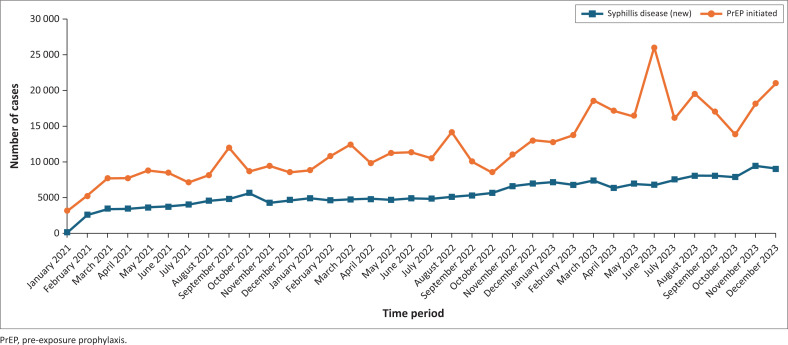
Syphilis cases and pre-exposure prophylaxis initiation in Zambian health facilities (rural and urban health facilities) from 2021 to 2023.

A monthly breakdown of the data was conducted to further explore the trends. As depicted in Figure 2, syphilis cases showed a consistent upward trend from 2021 to 2023, with notable surges observed in October 2021, during the period from November 2022 to March 2023, and reaching a peak in November 2023. In parallel, the number of individuals initiating PrEP also increased over the same period. Peaks in PrEP uptake were recorded in several months, including September 2021, March 2022, August 2022, March 2023, August 2023 and December 2023, with the most pronounced peak occurring in June 2023. This monthly analysis reveals clear periodic patterns that may help guide targeted interventions to manage both syphilis transmission and PrEP enrolment.

**FIGURE 3:**
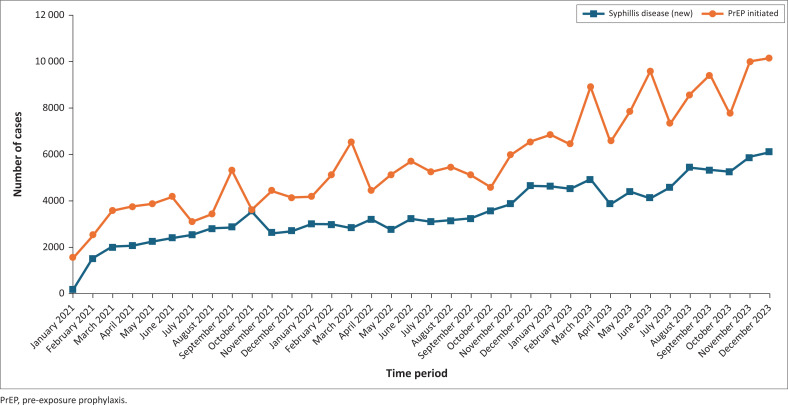
Syphilis cases and pre-exposure prophylaxis initiation in rural health facilities in Zambia from 2021 to 2023.

We also examined trends by comparing rural and urban facilities. As shown in Figure 3, rural facilities experienced a notable rise in STIs over the years, with an initial peak around October 2021, another surge between December 2022 and March 2023, and the most significant increase occurring from August 2023 to December 2023. In parallel, PrEP initiation in these rural areas also showed a steady upward trend between 2021 and 2023, culminating in its highest peaks in November 2023 and December 2023.

**FIGURE 4:**
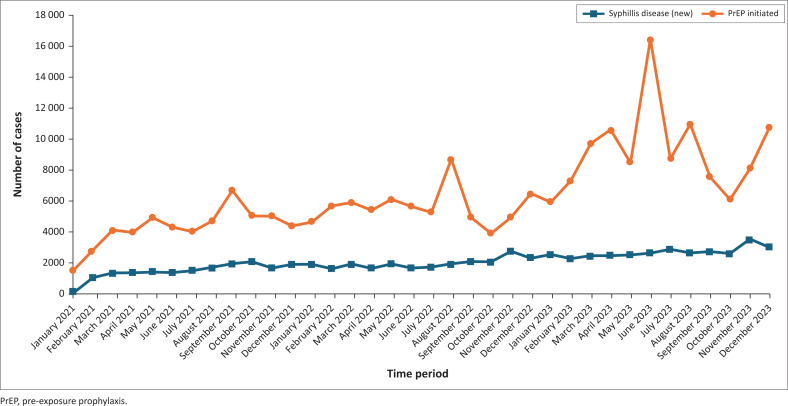
Incidence and pre-exposure prophylaxis uptake in urban health facilities in Zambia from 2021 to 2023.

Figure 4 illustrates the trends in syphilis cases and PrEP initiation within urban facilities over the 3-year period. Our analysis indicates that syphilis cases exhibited notable fluctuations throughout this time, with the highest number of cases occurring in November 2023, mirroring the trend seen in rural areas. However, the overall increase in syphilis cases was less pronounced in urban settings compared to their rural counterparts. In contrast, PrEP initiation displayed a more significant upward trend in urban facilities, reaching its peak in June 2023. These findings highlight the varying dynamics of syphilis trends and preventive interventions between urban and rural regions.

Following the descriptive analysis, we conducted a regression analysis of the national level data to investigate the association of the observed trends in syphilis cases and PrEP uptake. Our analysis found that there was a significant association between syphilis incidence and PrEP initiation with a coefficient value of 0.33. This means that with every unit increase in PrEP initiation, syphilis cases were expected to increase by 0.33, holding all other factors constant. This was statistically significant with a *p*-value of < 0.01 at 95% confidence interval (CI). Table 3 shows these results.

**TABLE 3:** Summary results of the linear regression.

Syphilis	Coef.	s.e.	*T*	*P*	95% CI
PrEP Initiation	0.33	0.04	8.38	0.00*	0.25–0.40
Constant	1561.19	509.17	3.07	0.04	556.44–2595.94

PrEP, pre-exposure prophylaxis; Coef., coefficient; s.e., standard error; 95% CI, confidence interval.

* , Significance level (*p* < 0.05).

## Discussion

Our findings indicate that the number of syphilis cases surged dramatically, more than doubling from 44 792 in 2021 to 91 583 in 2023. At the same time, PrEP initiation also experienced a significant increase, more than doubling to reach 210 195 during the same period. This parallel rise in both syphilis infections and PrEP uptake poses a major public health challenge. The interconnected nature of HIV and STI epidemics, a relationship recognised since the early days of the HIV crisis, further complicates the situation,^20,21^ and over the past years, STI cases have surged at a high rate.^11,14,22^

### Sexually transmitted infection incidence by rural and urban disaggregation

When disaggregated by rural and urban, this analysis shows that out of all the 199 567 syphilis cases reported across the 3 years, 63% were attributed to rural facilities compared to 37% of urban facilities. This trend was observed consistently over the 3 years. Despite this difference across rural and urban facilities, both these areas saw an increase in syphilis cases over the years.

Our findings align with those of a systematic review on STIs among PrEP users, which reported a pooled incidence of 72.2 per 100 person-years for a composite outcome of chlamydia, gonorrhoea and early syphilis during HIV pre-exposure prophylaxis.^7^

In rural areas, the higher incidence of syphilis cases could be related to limited prevention opportunities and resources in rural areas as compared to urban areas.^23^ In addition, overall knowledge of transmission of STIs is poor among rural populations as compared to their urban counterparts.^24^ This further demonstrates the vulnerability of rural populations and the need for targeted attention for rural facilities.

### Association of syphilis incidence and PrEP initiation

This analysis further found a statistically significant positive relationship between syphilis incidence and PrEP initiation, consistent with previous research showing higher STI rates in the 12 months following PrEP use compared to the preceding year (incidence rate ratios [IRR]: 1.72, CI: 1.22–2.41; aIRR: 1.39, CI: 0.98–1.96). In addition, PrEP users were found to be at a greater risk of STIs than post-exposure prophylaxis (PEP) users (IRR: 2.18, CI: 1.46–3.24; adjusted IRR: 1.76, CI: 1.14–2.71), with evidence suggesting that this increased risk may be associated with higher engagement in risky sexual behaviours among PrEP participants.^11^

Furthermore, recent studies have shown that the prevalence of syphilis at PrEP initiation increased from 7.8% to 13.3%.^25^ In a systematic review to investigate the burden of STIs among individuals using PrEP (emtricitabine and tenofovir disoproxil fumarate) for the prevention of HIV infection Ong and colleagues^7^ also found that studies reporting a combined measure of chlamydia, gonorrhoea and early syphilis showed a pooled prevalence of 23.9% at the start of HIV PrEP and a pooled incidence rate of 72.2 per 100 person-years during PrEP use. These findings highlighted the significant burden of STIs among both new and ongoing PrEP users.

The observed rise in STIs alongside the increase in PrEP initiation is likely linked to behavioural factors, as suggested by several studies. One study found that individuals often seek PrEP when engaging in higher-risk sexual behaviours, indicating that it is these behaviours, rather than PrEP use itself, that contribute to the increased incidence of STIs.^26^ Similarly, research conducted in Zimbabwe reported that following PrEP initiation, clients expressed increased confidence in their sexual relationships and reduced stress in negotiating condom use, which ultimately led to a decrease in condom use.^27^

Further evidence highlights that modifiable factors such as condom use, number of sexual partners, partner characteristics and healthcare-seeking behaviours play a critical role in STI acquisition.^28^ This growing body of evidence underscores the need for integrating STI screening and treatment services into all PrEP service delivery platforms to ensure that individuals at higher risk receive timely diagnosis and appropriate care.^13^

Although syphilis is easy to diagnose and treat, infection rates are still rising in Zambia, as reflected in our analysis. While the country continues to roll out the implementation of PrEP as the cornerstone of a combined prevention strategy for lowering the risk of new HIV infections, the WHO emphasises the need for integrating STI screening, diagnosis and treatment into PrEP programmes to enhance prevention efforts, improve health outcomes and strengthen public health interventions.^29^ Integrating rural STI risk reduction into a comprehensive framework could enable the targeted development of interventions tailored to the unique needs of rural communities.^23^

Furthermore, special attention must be focused on rural populations, acknowledging their vulnerability and challenges with accessing health services. We recommend that further detailed analysis be performed to investigate this association on patient-level data to account for several confounders.

### Strengths and limitations

The strength of our analysis is that it captured and utilised data from all 10 provinces of Zambia, allowing us to bring to light the national picture of syphilis incidence and PrEP uptake. In addition, the study used secondary data, which eliminated potential for biases that arise from primary data collection.

However, as our study utilised routinely collected programme data which are aggregated at facility level, this limited the analysis as other possible confounders that may influence the relationship between STI increase and PrEP uptake. Furthermore, we could not include data on the syphilis cases before the introduction of PrEP because of the unavailability of data on syphilis in the general population in DHIS2 at that time.

## Conclusion and recommendations

People who use PrEP for HIV prevention are also disproportionately affected by STIs, with syphilis being a notable concern. Although PrEP has proven highly effective in reducing the risk of HIV transmission, its widespread rollout may be inadvertently associated with an increase in syphilis cases, a trend that appears to be linked to various factors among PrEP users.

Our analysis suggests that there is a discernible association between the initiation of PrEP and a subsequent rise in syphilis infections, an observation that underscores the need for more detailed investigation into the underlying causes of this phenomenon. It is imperative to explore how changes in sexual behaviour, possibly prompted by a perceived lower risk of HIV, could be contributing to this surge in syphilis cases, thereby necessitating a closer look at the sexual practices of PrEP users and the potential behavioural factors that may be driving these trends. The implications of these findings are significant, indicating that an integrated approach to managing HIV and STIs is critical for the overall effectiveness of public health interventions.

In response to these observations, we recommend that enhancing surveillance systems to monitor both syphilis incidence and PrEP uptake is vital; such systems would enable early detection of trends and potential outbreaks, thereby facilitating a more proactive response in the provision of comprehensive care. This dual focus on enhanced data collection and analysis is expected to contribute significantly to the early identification and management of syphilis cases, ensuring that public health responses remain agile and responsive to evolving patterns in sexual health.

In parallel, we recommend further analytical efforts to understand the broader context of sexual behaviour among PrEP users. Such analysis should aim to uncover the specific practices and circumstances that might be influencing the increased incidence of syphilis and should be designed to provide insights that will inform the development of targeted, appropriate interventions.

Moreover, this study highlights the critical need for enhanced syphilis prevention strategies within the context of PrEP programmes. These strategies should encompass a range of interventions, including comprehensive counselling, which would provide PrEP users with the necessary information and support to make informed decisions about their sexual health. Regular and systematic testing for syphilis should be integrated into routine PrEP care protocols to ensure that any new infections are detected and managed promptly.

In addition, healthcare services need to be tailored specifically to the needs of PrEP users, recognising that this population may have unique risk profiles and requirements that differ from the general population. By aligning syphilis prevention efforts with existing PrEP services, public health initiatives can achieve a more holistic approach to managing sexual health, effectively addressing the dual challenges of HIV and syphilis.

Ultimately, it is crucial for public health policies to reflect these concerns by ensuring that interventions for syphilis prevention are not implemented in isolation but rather are integrated seamlessly with PrEP services. Such integration would help manage and reduce the incidence of syphilis more effectively, thereby reinforcing the overall public health response to STIs. By fostering a coordinated strategy that combines improved surveillance, targeted behaviour analysis and comprehensive prevention measures, the healthcare system can better safeguard the sexual health of PrEP users and contribute to a more effective overall reduction in the burden of both HIV and syphilis.
